# Risk of Parkinson’s disease following gout: a population-based retrospective cohort study in Taiwan

**DOI:** 10.1186/s12883-020-01916-9

**Published:** 2020-09-08

**Authors:** Li-Yu Hu, Albert C. Yang, Shyh-Chyang Lee, Zi-Hong You, Shih-Jen Tsai, Chang-Kuo Hu, Cheng-Che Shen

**Affiliations:** 1 Department of Psychiatry, Taipei Veterans General Hospital, Taipei, Taiwan; 2 School of Medicine, National Yang-Ming University, Taipei, Taiwan; 3 Department of Orthopedics, Chiayi Branch, Taichung Veterans General Hospital, Chiayi, Taiwan; 4 Division of Nephrology, Department of Internal Medicine, Chiayi Branch, Taichung Veterans General Hospital, Chiayi, Taiwan; 5 Division of Neurosurgery, Department of surgery, Chiayi Branch, Taichung Veterans General Hospital, No. 600, Sec. 2, Shixian Rd., West District, Chiayi City, Taiwan; 6 Department of Psychiatry, Chiayi Branch, Taichung Veterans General Hospital, No. 600, Sec. 2, Shixian Rd., West District, Chiayi City, Taiwan; 7 Center for Innovative Research on Aging Society (CIRAS), National Chung Cheng University, Chiayi, Taiwan

**Keywords:** Gout, National Health Insurance Research Database, Parkinson disease

## Abstract

**Background:**

The progressive neurodegenerative disorder Parkinson disease (PD) is well-established as the second most common neurodegenerative disease. Associations between the sequential risk of PD and gout have been addressed in other studies, but findings have been inconclusive. Accordingly, we executed the present study with the purpose of assessing PD risk in patients with gout.

**Methods:**

From Taiwan’s National Health Insurance Research Database, we identified the data of patients newly diagnosed as having gout between January 1, 2000 and December 1, 2000. A cohort of patients without gout, matched for sex and age, was constructed for comparison. Hazard ratios (HRs) and the incidence rate of subsequent PD were calculated for both cohorts and separately for male and female groups. The gout and comparison cohorts consisted of 7900 patients each.

**Results:**

The HR for PD was not significantly higher in the gout cohort compared with the control cohort (HR 1.01, 95% confidence interval [CI], 0.93–1.31, *P* = .268), even after adjustment for age, urbanization, monthly income, sex, and comorbidities. We did not observe gender differences in the gout–PD association (male: HR 1.01, 95% CI, 0.88–1.36, *P* = .400; female: HR 1.11, 95% CI, 0.84–1.46, *P* = .466).

**Conclusions:**

Our study identified that there was no protective effect of gout for the risk of PD in the Taiwanese population.

## Background

The well-known progressive neurodegenerative disorder Parkinson disease (PD) involves dopaminergic nigrostriatal neuron degeneration, which typically results in motor deficits. It has an estimated worldwide prevalence of 1 to 2% for individuals over the age of 65 years, rendering it the second most common neurodegenerative disease, with the most common being Alzheimer disease [1]. This disorder’s global burden has increased by over two times over the past generation because of the increase in the number of elderly individuals [2]. An epidemiology survey has revealed that the PD incidence and prevalence are higher in Western populations than in Asians, including in the Taiwanese people [3, 4]. However, no definite evidence could explain geographic differences until now. There has been considerable progress in determining the environmental and genetic factors that influence the development of PD. Additionally, accumulating evidence indicates oxidative stress to play a major role in the PD etiology [5, 6].

Uric acid (UA) has been demonstrated by previous research to have an antioxidative effect [7–9]. Due to its antioxidant activity, UA was thought to protect neuronal cells, consequently playing a possibly protective role in neurodegenerative diseases. For example, studies have revealed that patients with multiple sclerosis (MS) are known to present lower serum UA levels whereas gout patients have much lower risk for developing MS [10]. Although the role of uric acid in the pathogenesis of PD has not been fully elucidated, there have been several studies demonstrating the associations between the serum UA concentrations [11, 12] and the risk of developing PD or the severity of the progressive symptoms in PD and Alzheimer’s disease [13]. Findings reported by previously executed research have been inconsistent with respect to the gout–PD risk association, despite some of such research being large retrospective studies [14–19], as shown in Table 1. For example, an English study found that in the gout group, the increase in the risk of subsequent PD was modest (risk ratio 1.11, 95% confidence interval [CI], 1.05–1.17) [15]. However, another study in the United Kingdom used a large population-based database and concluded that the risk of PD development is relatively low in individuals having a gout history (risk ratio 0.69, 95% CI, 0.48–0.99). In addition, other studies have demonstrated variations in PD incidence regarding ethnicity and race [20, 21]. Asian studies on gout and PD risk are, however, few [18].

**Table 1:** Summary of Studies used to estimate the Risk of Parkinson’s Disease among the Patients with Gout

Study	Year	Study Design	Ethnic Group (Country)	Risk Ratio (95% CI^**a**^)
Alonso et al.	2007	Case-control	Europe (United Kingdom)	0.69 (0.48–0.99)
De Vera et al.	2008	Retrospective cohort	North America (Canada)	0.70 (0.59–0.83)
Schernhammer et al.	2013	Case-control	Europe (Denmark)	1.06 (0.90–1.25)
Lai et al.	2014	Case-control	Asia (Taiwan)	1.00 (0.90–1.11)
Pakpoor et al.	2015	Retrospective cohort	Europe (United Kingdom)	1.11 (1.05–1.17)
The present study	2020	Retrospective cohort	Asia (Taiwan)	1.01 (0.93–1.31)

^a^CI indicates confidence interval

To address the previously described inconsistencies in findings regarding the gout–PD risk association and the paucity of Asian studies concerning said association, we executed the present nationwide population-based retrospective cohort study to seek to identify whether an association exists between the two aforementioned illnesses. The following hypothesis constituted the basis for our study: patients with gout would have a relatively low risk of PD.

## Methods

### Data sources

Taiwan’s 1995-established National Health Insurance (NHI), covering approximately 99% of residents of Taiwan, is a compulsory program covering comprehensive medical care, such as emergency, outpatient, inpatient, and traditional Chinese medicine services [22]. The National Health Insurance Research Database (NHIRD), which is overseen by the National Health Research Institutes, comprises exhaustive information concerning clinic visits, including prescription details as well as diagnostic codes based on the A code and International Classification of Disease, Ninth Revision, Clinical Modification (ICD-9-CM). For this database, data confidentiality is subject to the directives of the NHI Bureau. For executing the present study, we retrieved Longitudinal Health Insurance Database 2000 (LHID2000) data; the LHID2000 comprises the information of 1 million people who were randomly and systematically sampled from the NHIRD. Comparing patients whose information is contained in the NHIRD and those whose information is contained in the LHID2000 demonstrated no significant differences with regard to average distribution of sex or age or the amount of insured payroll [23].

### Ethics statement

The Institutional Review Board (IRB) of Taipei Veterans General Hospital ratified our executed study (IRB number: 2018–07-016 AC). Because the NHIRD comprises secondary data that had been deidentified and maintained for research purposes, written consent from the patients evaluated in this study was unnecessary. Accordingly, the aforementioned IRB provided a formal written waiver of the requirement to obtain patients’ written consent.

### Study population

From the LHID2000, we included the data of patients aged more than 20 years and older who received a new diagnosis of gout between January 1, 2000 and December 31, 2000. Gout was defined according to ICD-9-CM code 274. To ensure patient homogeneity and diagnostic validity, we enrolled patients only if they had at least two consensus diagnoses of gout during the observational period. Patients diagnosed as having PD before enrollment (A code: A221; ICD-9-CM code: 332) were excluded. Moreover, for each gout patient included in our final cohort, we randomly selected from the LHID2000 the data of a control patient (i.e., a patient not diagnosed as having gout or PD) matched for age, enrollment date, and sex. Observations of all control patients and patients with gout were started since the enrollment date until (1) PD diagnosis by a neurologist, (2) death, or (3) the end date, December 31, 2013. Neurologist-diagnosed PD constituted our primary clinical outcome.

### Statistical analysis

By applying independent *t* and chi-squared tests, we probed differences between demographic characteristics of patients with gout and control patients. The incidence of newly diagnosed PD in the patient groups was also calculated after stratification of the data by age (≥65 or < 65 years), sex, and time since gout diagnosis.

By executing Cox proportional hazards regression, we identified variables predicting PD in the two groups. Many control variables were included in the univariate analysis as covariates; these were sex, age, urbanization, typical comorbidities (diabetes mellitus, chronic liver disease, dyslipidemia, cerebrovascular disease, hypertension, autoimmune disease, nephropathy, and chronic lung disease), and monthly income. We estimated patients’ monthly income by using their insurance premiums, typically derived on the basis of the beneficiary’s total income. We categorized the estimated monthly income the following groups: no income, low (< 625 US Dollar), medium (> 625 US Dollar to < 1250 US Dollar), and high (≥1250 US Dollar). In addition, we divided urbanization into the following categories: urban, suburban, and rural. Then, we included univariate analysis factors with a moderate statistically significant relationship (i.e., *P* < .1) into a multivariate Cox proportional-hazards regression model by using the forward selection method [24]. Variables predicting PD in male and female groups separately were also identified using the same regression.

For executing data extraction and computation, we employed Perl (Version 5.12.2). Furthermore, we executed data linkage and processing as well as control sampling through Microsoft SQL Server 2005(Microsoft Corp., Redmond, WA, USA). All statistical analysis processes were completed using SPSS (Version 19.0 for Windows; IBM Corp., New York, NY, USA) and SAS (Version 9.2; SAS Institute Inc., Cary, NC, USA). We deemed *P* < .05 as signifying statistical significance.

## Results

### Participant selection

Of the 7900 control individuals and 7900 patients with gout, 83.9% were determined to be men. We noted the median (interquartile range [IQR]) age at the time of enrollment to be 50 (40–64) years; in addition, we observed the median (IQR) follow-up periods for patients with gout and controls to be 13.36 (13.04–13.65) and 13.36 (13.04–13.63) years, respectively. Patients with gout more frequently had comorbidities such as hypertension, cerebrovascular disease, diabetes mellitus, dyslipidemia, nephropathy, autoimmune disease, chronic lung disease, and chronic liver disease than did controls. Clinical and demographic variables of patients with gout and controls are shown in Table 2.

**Table 2:** Baseline characteristics of patients with and without gout

Demographic data	Patients with Gout *n* = 7900		Patients without Gout *n* = 7900		*P* value
	*n*	%	*n*	%	
Age (years)^a^	50 (40–64)		50 (40–64)		
≥ 65	1791	11.5	1791	11.5	.999
< 65	6109	88.6	6109	88.6	
Sex					
Male	5409	83.9	5409	83.9	.999
Female	2491	16.1	2491	16.1	
Comorbidities					
Diabetes mellitus	1664	9.6	950	4.5	<.001*
Hypertension	3240	24.3	1887	9.8	<.001*
Dyslipidemia	2230	14.0	1000	4.8	<.001*
Cerebrovascular disease	1147	6.9	829	3.7	<.001*
Chronic lung disease	989	4.9	684	1.9	<.001*
Nephropathy	1219	5.9	661	2.8	<.001*
Chronic liver disease	2713	19.4	1638	8.7	<.001*
Autoimmune disease	316	2.2	163	1.2	<.001*
Degree of urbanization					<.001*
Urban	4186	56.9	4552	62.2	
Suburban	2853	35.2	2694	32.0	
Rural	861	7.9	654	5.8	
Income group					.023*
High income	984	15.6	1007	17.0	
Medium income	1314	49.2	1261	50.4	
Low income	4184	19.9	4073	17.8	
No income	1418	15.4	1559	14.8	
Follow-up years^a^	13.36 (13.04–13.65)		13.36 (13.04–13.63)		.005*

^a^ indicates Median (interquartile range); * indicates statistical significance

### Incidence of PD in gout and control cohorts

We determined that during the study period, 247 controls (2.63 per 1000 person-years) and 339 patients with gout (3.56 per 1000 person-years) received a PD diagnosis. Between the patients with gout and controls, the PD rate ratio (RR) was 1.36 (95% CI, 1.15–1.60, *P* < .001), with the RR remaining higher in patients with gout than in controls after stratification for age (≥65 or < 65 years) and sex (male or female) (Table 3). For stratification by follow-up duration (0–1, 1–5, ≥5 years), the RR of newly diagnosed PD was determined to remain significantly higher in the patients with gout.

**Table 3:** Incidence of Parkinson Disease in Patients with and without Gout

	Patients with Gout		Patients without Gout		Rate ratio (95% CI)	*P* value
	No. of Parkinson Disease	Per 1000 person-years	No. of Parkinson Disease	Per 1000 person-years		
Total	339	3.56	247	2.63	1.36 (1.15–1.60)	<.001*
Age						
≥ 65	214	12.37	159	9.31	1.33 (1.08–1.64)	.006*
< 65	125	1.60	88	1.14	1.40 (1.06–1.86)	.015*
Sex						
Male	204	3.13	154	2.41	1.30 (1.05–1.62)	.013*
Female	135	4.49	93	3.10	1.45 (1.11–1.91)	.006*
Follow-up						
0–1	33	702.13	17	257.58	2.73 (1.48–5.22)	<.001*
1–5	90	62.54	65	37.06	1.69 (1.21–2.36)	<.001*
≥ 5	216	2.30	165	1.79	1.29 (1.05–1.59)	.014*

CI indicates confidence interval; * indicates statistical significance

**Table 4:** Analyses of risk factors for Parkinson Disease in patients with and without Gout

Predictive variables	Univariate analysis		Multivariate analysis	
	HR (95% CI)	*P* value	HR (95% CI)	*P* value
Gout	1.36 (1.15–1.60)	<.001	1.01 (0.93–1.31)	.268
Age (< 65 = 0, ≥65 = 1)	8.20 (6.93–9.71)	<.001	4.41 (3.61–5.39)	<.001*
Sex (Male = 0, Female = 1)	1.37 (1.16–1.62)	<.001	0.90 (0.76–1.08)	.256
Comorbidities				
Diabetes mellitus	2.83 (2.38–3.36)	<.001	1.32 (1.08–1.60)	.006*
Hypertension	4.62 (3.90–5.48)	<.001	1.85 (1.52–2.27)	<.001*
Dyslipidemia	2.01 (1.69–2.38)	<.001	0.90 (0.74–1.09)	.281
Cerebrovascular disease	4.63 (3.91–5.48)	<.001	2.04 (1.69–2.45)	<.001*
Chronic lung disease	2.89 (2.38–3.52)	<.001	1.25 (1.02–1.53)	.035*
Nephropathy	2.16 (1.77–2.63)	<.001	1.07 (0.86–1.32)	.563
Chronic liver disease	1.80 (1.53–2.13)	<.001	1.23 (1.02–1.47)	.026*
Autoimmune disease	1.66 (1.13–2.42)	.009	1.19 (0.81–1.75)	.369
Degree of urbanization				
Urban	Reference		Reference	
Suburban	1.34 (1.12–1.59)	.001	1.11 (0.92–1.33)	.276
Rural	2.00 (1.57–2.54)	<.001	1.32 (1.02–1.71)	.033*
Income group				
High income	Reference		Reference	
Medium income	5.51 (3.59–8.44)	<.001	1.90 (1.21–2.99)	.005*
Low income	3.94 (2.60–5.95)	<.001	0.98 (1.04–2.47)	.033*
No income	1.30 (0.26–2.54)	.304	1.26 (0.76–2.10)	.369

*HR* Indicates hazard ratio, *CI* Indicates confidence interval; * indicates statistical significance

### Gout and PD risk

Compared with controls, the gout cohort did not have a significantly higher hazard ratio (HR) for PD development during the follow-up period (HR 1.01, 95% CI, 0.93–1.31, *P* = .268), even after adjustment for sex, age, urbanization, monthly income, and comorbidities (Table 4). After stratification by sex, the HR for PD was not significantly higher among patients with gout than among controls (HR 1.01, 95% CI, 0.88–1.36, *P* = .400; HR 1.11, 95% CI, 0.84–1.46, *P* = .466) (Supplemental Tables 1 and 2).

## Discussion

Results indicate that the RR of newly diagnosed PD was significantly increased in the gout cohort relative to the control cohort in our study. In this study’s gout cohort, the HR for PD was not significantly higher than that in the control cohort. Patients with gout more frequently had hypertension, cerebrovascular disease, diabetes mellitus, nephropathy, dyslipidemia, autoimmune disease, chronic liver disease, and chronic lung disease than did controls. Other previously executed studies have also demonstrated an association between gout and various metabolic and cardiovascular diseases [25–27]. In addition, no gender difference was evident in the gout–subsequent PD risk association.

Results of our study fail to demonstrate an association between gout and PD, even though many studies have determined that UA has an antioxidative effect [7–9]. The possible explanation for this finding is twofold. First, gout is determined to be associated with metabolic and cardiovascular diseases [25, 26]; as demonstrated by previously executed research, these diseases constitute independent risk factors for PD [28, 29]. For example, a prospective study of the Finnish population revealed that the HR of PD among individuals with type 2 diabetes relative to those without the disease was 1.85 (95% CI, 1.23–2.80) [30]. The increased prevalence of metabolic diseases may offset the protective effect afforded by UA against the sequential PD risk in patients with gout. The finding of a higher frequency of hypertension, cerebrovascular disease, dyslipidemia, and diabetes mellitus in this study’s gout cohort compared with the control cohort supports this explanation. Second, studies have shown an association between gout, a chronic inflammatory disease, and high levels of various inflammatory cytokines, chiefly interleukin (IL)-1, IL-6, and IL-8 [27, 31]. Additionally, although accumulating research evidence suggests the mechanisms of the inflammation in the pathogenesis of PD were far more complicated [32, 33] than the assumptions made by previous studies [34, 35], the vast majority of studies still support the idea that inflammation plays a prominent role in mediating the progressive neurodegeneration in PD [36]. Thus, the protective effect of UA in patients with gout may be negated under the influences of the inflammation.

The relationship between gout and PD risk has been evaluated in some retrospective studies, which have yielded inconsistent results [14–19]. A modestly increased risk of subsequent PD in the gout group was observed in one of the studies [15]. However, a decreased risk of PD was found for patients with gout in two other studies [16, 17]. Similar to our study result, two studies failed to identify an association between gout and PD [18, 19]. Different study designs may partially account for the conflicting results. Furthermore, the question as to whether genetic differences along racial or ethnic lines influence PD risk in patients with gout warrants consideration because previously executed research has revealed that race and ethnicity have an influence on the incidence of PD [20, 21].

Risk factors for PD have been found to be gender specific [37–39]. For example, one meta-analytic study determined an association between aspirin use by men but not by women with an increased PD risk (men: RR 1.22, 95% CI, 1.03–1.44; women: RR 0.98, 95% CI, 0.71–1.37) [38]. With respect to gout and the subsequent risk of PD, one previous study also demonstrated a gender specific difference, finding a decreased PD risk among men with gout (OR 0.60, 95% CI, 0.40–0.91) but not among women with gout (OR 1.26, 95% CI, 0.57–2.81) [17]. However, in our study, no gender difference was identified regarding the gout–subsequent PD association (Supplemental Tables 1 and 2). Additional studies are required to identify whether the gout–subsequent PD risk association is affected by gender.

Our study has two main strengths: large sample size and specialist-executed PD diagnoses. Moreover, the patient selection process was unbiased. Due to compulsory enrollment in NHI and easy access to low-cost health care, the patient population exhibits low referral biases and high follow-up compliance. However, we must discuss our study’s limitations. First, the NHIRD does not have information regarding a family history of PD, environmental factors (such as exposure to herbicides or pesticides), or lifestyle factors (such as coffee or tobacco consumption), but all of these may be associated with PD risk [40–43]. Second, NHIRD-using studies could not obtain records of serum UA levels; accordingly, further research should be performed on whether serum UA levels affect PD risk. Third, this study’s follow-up period may have been inadequate for the detection of late-onset PD. Hence, future research must include a longer follow-up period to clarify the long-term risk of PD in patients with gout. Finally, in NHI claims, the indicated diagnoses are primarily for administrative billing purposes; they are not subject to verification for scientific purposes. Accordingly, the manner in which diagnoses were classified remains unclear in studies using the NHIRD. Thus, we cannot determine the accuracy of the diagnoses.

## Conclusion

In conclusion, no gout–PD association was found in this nationwide retrospective cohort study of patients in the Taiwanese population. We did, however, identify further evidence of associations between gout and metabolic and cardiovascular diseases. Population-based prospective studies in the future should include longer follow-up periods to further probe the gout–PD risk association in different racial or ethnic groups.

## Supplementary information

**Additional file 1:** **Supplemental 1.** Analyses of Risk Factors for Parkinson Disease in Male Patients with and withoutGout. **Supplemental 2.** Analyses of Risk Factors for Parkinson Disease in Female Patients with and withoutGout.

## Data Availability

The data that support the findings of this study are available from the Taiwan National Health Insurance Research Database (NHIRD). Interested individuals should contact the NHIRD to gain access.

## Abbreviations

PD: Parkinson’s disease
UA: Uric acid
CI: Confidence interval
NHI: National Health Insurance
NHIRD: National Health Insurance Research Database
ICD-9-CM: International Classification of Disease, Ninth Revision, Clinical Modification
LHID2000: Longitudinal Health Insurance Database 2000
IRB: Institutional Review Board
IQR: Interquartile range
RR: Rate ratio
HR: Hazard ratio
IL: Interleukin
RR: Relative risk
