# Supplementary material for: Risk of Parkinson’s disease following gout: a population-based retrospective cohort study in Taiwan
Source: BMC Neurol. 2020 Sep 8;20:338. doi: 10.1186/s12883-020-01916-9 (PMC7487828; doi:10.1186/s12883-020-01916-9)
Supplement: Supplementary file 1 — Additional file 1: Supplemental 1. Analyses of Risk Factors for Parkinson Disease in Male Patients with and withoutGout. Supplemental 2. Analyses of Risk Factors for Parkinson Disease in Female Patients with and withoutGout. [file 12883_2020_1916_MOESM1_ESM.docx]

**Supplemental 1 Analyses of Risk Factors for Parkinson Disease in Male Patients with and withoutGout**

| Predictive variables | | Univariate analysis | | | Multivariate analysis | | |
| --- | --- | --- | --- | --- | --- | --- | --- |
|  |  | HR (95% CI) | *P* value | | HR (95% CI) | *P* value | |
| Gout | | 1.30 (1.06–1.60) | .014 | 1.01 (0.88–1.36) | | .400 |  |
| Age(<65 = 0, ≥65 = 1) | | 10.20 (8.20–12.68) | <.001 | 5.28 (4.04–6.90) | | <.001* |  |
| Comorbidities | |  |  |  | |  |  |
|  | Diabetes mellitus | 2.90 (2.30–3.66) | <.001 | 1.27 (0.97–1.65) | | .078 |  |
|  | Hypertension | 4.89 (3.95–6.04) | <.001 | 1.81 (1.40–2.33) | | <.001* |  |
|  | Dyslipidemia | 1.86 (1.47–2.36) | <.001 | 0.83 (0.64–1.09) | | .175 |  |
|  | Cerebrovascular disease | 5.36 (4.30–6.68) | <.001 | | 2.12 (1.67–2.70) | <.001* | |
|  | Chronic lung disease | 3.51 (2.76–4.47) | <.001 | | 1.27 (0.99–1.65) | .064 | |
|  | Nephropathy | 2.20 (1.68–2.90) | <.001 | | 0.91 (0.68–1.22) | .537 | |
|  | Chronic liver disease | 1.75(1.41–2.17) | <.001 | | 1.23 (0.98–1.56) | .078 | |
|  | Autoimmune disease | 1.93 (1.15–3.23) | .013 | | 1.24 (0.74–2.09) | .420 | |
| Degree of urbanization | |  |  | |  |  | |
|  | Urban | Reference |  | | Reference |  | |
|  | Suburban | 1.34 (1.07–1.68) | .010 | | 1.15 (0.91–1.45) | .246 | |
|  | Rural | 2.08 (1.53–2.84) | <.001 | | 1.46 (1.05–2.02) | .023* | |
| Income group | |  |  | |  |  | |
|  | High income | Reference |  | | Reference |  | |
|  | Medium income | 5.40 (3.37–8.66) | <.001 | | 1.73 (1.05–2.86) | .032* | |
|  | Low income | 3.72 (2.40–5.75) | <.001 | | 1.42 (0.89–2.27) | .137 | |
|  | No income | 1.08 (0.61–1.92) | .792 | | 1.05 (0.59–1.87) | .863 | |

HR indicates hazard ratio; CI indicates confidence interval; * indicates statistical significance.

**Supplemental 2Analyses of Risk Factors for Parkinson Disease in Female Patients with and withoutGout**

| Predictive variables | | Univariate analysis | | | Multivariate analysis | | |
| --- | --- | --- | --- | --- | --- | --- | --- |
|  |  | HR (95% CI) | *P* value | | HR (95% CI) | *P* value | |
| Gout | | 1.45 (1.11–1.89) | <.001 | 1.11 (0.84–1.46) | | .466 |  |
| Age(<65 = 0, ≥65 = 1) | | 5.56 (4.25–7.28) | <.001 | 3.50 (2.59–4.74) | | <.001* |  |
| Comorbidities | |  |  |  | |  |  |
|  | Diabetes mellitus | 2.54 (1.95–3.31) | <.001 | 1.38 (1.03–1.86) | | .033* |  |
|  | Hypertension | 4.04 (3.02–5.40) | <.001 | 1.90 (1.37–2.64) | | <.001* |  |
|  | Dyslipidemia | 2.03 (1.56–2.63) | <.001 | 0.99 (0.73–1.33) | | .945 |  |
|  | Cerebrovascular disease | 3.55 (2.72–4.64) | <.001 | | 1.91 (1.44–2.54) | <.001* | |
|  | Chronic lung disease | 2.03 (1.45–2.84) | <.001 | | 1.17 (0.83–1.64) | .377 | |
|  | Nephropathy | 1.94 (1.44–2.60) | <.001 | | 1.26 (0.93–1.72) | .143 | |
|  | Chronic liver disease | 1.82(1.40–2.37) | <.001 | | 1.23 (0.92–1.63) | .158 | |
|  | Autoimmune disease | 1.29 (0.74–2.25) | .376 | |  |  | |
| Degree of urbanization | |  |  | |  |  | |
|  | Urban | Reference |  | | Reference |  | |
|  | Suburban | 1.31 (0.99–1.74) | .059 | | 1.08 (0.81–1.46) | .594 | |
|  | Rural | 1.81 (1.23–2.65) | .002 | | 1.20 (0.79–1.82) | .392 | |
| Income group | |  |  | |  |  | |
|  | High income | Reference |  | | Reference |  | |
|  | Medium income | 6.85 (1.69–27.81) | .007 | | 2.52 (0.61–10.38) | .200 | |
|  | Low income | 5.26 (1.30–21.30) | .020 | | 2.15 (0.52–8.81) | .289 | |
|  | No income | 2.32 (0.53–10.16) | .263 | | 2.04 (0.47–8.94) | .343 | |

HR indicates hazard ratio; CI indicates confidence interval; * indicates statistical significance.
